# A Prothrombotic Shift: Cerebral Venous Sinus Thrombosis in the Recovery Phase of Dengue Fever—A Case Report

**DOI:** 10.1002/ccr3.72819

**Published:** 2026-05-26

**Authors:** Zain Ul Abedeen, Waqar Khan, Waqar Iqbal Khan, Zia Ullah, Naeem Ullah, Abad Ur Rehman

**Affiliations:** ^1^ Department of Medicine Saidu Medical College Swat Pakistan; ^2^ Department of Medicine Spinghar University (Kabul Campus) Kabul Afghanistan

**Keywords:** case report, cerebral venous sinus thrombosis, dengue fever, prothrombotic state, thrombocytopenia

## Abstract

Post‐dengue cerebral venous sinus thrombosis is an uncommon complication of dengue fever. It occurs due to a prothrombotic shift during the recovery or critical phase of dengue fever, presenting usually as a persistent headache. Prompt neuroimaging and anticoagulation are crucial to a favorable prognosis, even in the presence of hemorrhagic infarction.

**Abbreviations**
AHA: American Heart Association
ALP: alkaline phosphatase
ALT: alanine aminotransferase
ASA: American Stroke Association
AST: aspartate aminotransferase
BUN: blood urea nitrogen
CT: computed tomography
CVST: cerebral venous sinus thrombosis
EAN: European Academy of Neurology
EF: ejection fraction
eGFR: estimated glomerular filtration rate
ESO: European Stroke Organization
GCS: Glasgow Coma Scale
ICP: intracranial pressure
MCV: mean corpuscular volume
MRI: magnetic resonance imaging
MRV: magnetic resonance venography
NS1: non‐structural protein 1
Q12H: every 12 hours (*Quaque 12 Hora*)
Q8H: every 8 hours (*Quaque 8 Hora*)
TTE: transthoracic echocardiography
WBCs: white blood cells

## 1 Introduction

Dengue fever is a vector‐borne viral infection caused by a flavivirus and spreads to humans through the bites of infected *Aedes* mosquitoes. It is endemic in tropical and subtropical regions, infects an estimated 400 million people annually, and represents a major burden on global health in terms of both illness and death [1]. Clinically, dengue demonstrates a broad spectrum ranging from mild, self‐limiting febrile illness to severe disease marked by plasma leakage, hemorrhagic manifestations, and multi‐organ dysfunction [2].

Neurological complications associated with dengue are heterogeneous, including meningitis, encephalitis, acute disseminated encephalomyelitis, encephalopathy, Parkinsonism, neuro‐ophthalmic manifestations, cranial neuropathies, transverse myelitis, Guillain–Barré syndrome, and cerebrovascular events such as intracranial hemorrhage or thrombotic episodes [3]. While hemorrhagic manifestations remain the hallmark of severe dengue due to thrombocytopenia and capillary leak, thrombotic complications are a paradoxical and underrecognized phenomenon. Proposed mechanisms for this paradoxical prothrombotic state include virus‐induced endothelial dysfunction, immune‐mediated vascular injury, hemoconcentration, and transient hypercoagulability during the critical or recovery period [4].

Cerebral venous sinus thrombosis (CVST) is an uncommon but potentially life‐threatening form of cerebrovascular disorder caused by thrombosis of the dural venous sinuses. It accounts for 1% of all strokes and is seen more often in younger patients, with a higher incidence in females [5]. CVST is associated with a broad spectrum of inherited and acquired conditions that promote a prothrombotic state, including thrombophilias, malignancy, hormonal influences, infections, trauma, vasculitis, hematological abnormalities, systemic disorders, and certain medications. However, its occurrence in the direct temporal setting of a recent dengue fever is highly uncommon but has been increasingly documented in recent years in clinical case registries [6].

Hereby, we report a case of CVST occurring in temporal association with dengue infection, emphasizing the importance of maintaining a high index of clinical suspicion of thrombotic complications in patients presenting with persistent or atypical neurological symptoms during or following dengue illness. The case adds to the existing literature on thrombotic complications occurring concomitant with dengue and carries implications for clinicians and healthcare workers.

## 2 Case Report

### 2.1 Patient History and Examination

A 29‐year‐old South Asian male presented with a complaint of headache (day 11), which started 3 days ago. The pain was initially mild, localized to the frontal region, but progressive in nature and later became holocranial. It was not responding to the routine oral analgesics, relieved on lying down, and worsened with movement. It was also associated with frequent vomiting episodes prior to admission, with no further episodes during the inpatient stay. The patient also complained of abnormal sensitivity to light and dizziness. There was no fever, tinnitus, or aura.

The patient had a history of dengue fever, with symptoms starting 11 days back (day 1). The diagnosis of dengue was considered based on the presentation of patient symptoms, endemic occurrence, and decreasing platelets, and was confirmed by a positive NS1 [7] antigen test. The patient had received symptomatic treatment, showed signs of improvement, and transitioned smoothly into the recovery phase. There was no reported evidence of severe hemorrhagic complications, mucosal bleeding, or hemodynamic shock. His platelets were 94 × 10^8^/L and 111 × 10^8^/L, checked 11 (day 1) and 6 (day 5) days ago, respectively.

On the general inspection, the patient was alert and oriented (GCS = 15/15). The patient's vitals were stable: blood pressure was 100/80 mmHg, pulse rate was 79 beats per minute, and oxygen saturation was 97%. Neurological examination revealed equal and reactive pupils, normal cerebellar functioning, no focal neurological deficits, and no signs of meningismus. Fundoscopic examination was also normal.

### 2.2 Methods (Differential Diagnosis, Investigations, and Treatment)

The routine laboratory investigations were ordered after clinical evaluation (day 11). A complete blood count showed hemoglobin of 159 g/L, a platelet count of 135 × 10^8^/L, and a total leukocyte count of 9.39 × 10^9^/L. Liver function tests showed elevated alanine transaminase (ALT 107 U/L) and elevated aspartate transaminase (AST 77 U/L), with normal alkaline phosphatase (60 U/L). Total serum bilirubin was measured at 15.4 μmol/L. Renal function tests showed serum urea 5.7 mmol/L, blood urea nitrogen (BUN) 2.5 mmol/L, serum creatinine 74.3 μmol/L, and estimated glomerular filtration rate (eGFR) 116 mL/min/1.73 m^2^. The bleeding profile revealed a bleeding time of 0.9 min. A summary of these investigations has been presented in Table 1.

**TABLE 1:** Laboratory investigations of the patient.

Test	Patient value	Reference range
Hemoglobin	159 g/L	125–175 g/L
MCV	85 fL	78–98 fL
WBCs	9.39 × 10^9^/L	4–11 × 10^9^/L
Platelets	135 × 10^9^/L	150–400 × 10^9^/L
ALT	107 U/L	10–40 U/L
AST	77 U/L	5–45 U/L
ALP	60 U/L	40–390 U/L
Serum total bilirubin	15.4 μmol/L	5–25.7 μmol/L
Urea	5.7 mmol/L	0.8–7.5 mmol/L
BUN	2.5 mmol/L	2.5–8.6 mmol/L
Creatinine	74.3 μmol/L	53–106 μmol/L
eGFR	116 mL/min per 1.73 m^3^	> 90 mL/min per 1.73 m^3^

Abbreviations: ALP, alkaline phosphatase; ALT, alanine aminotransferase; AST, aspartate aminotransferase; BUN, blood urea nitrogen; eGFR, estimated glomerular filtration rate; MCV, mean corpuscular volume; WBCs, white blood cells.

**FIGURE 1:**
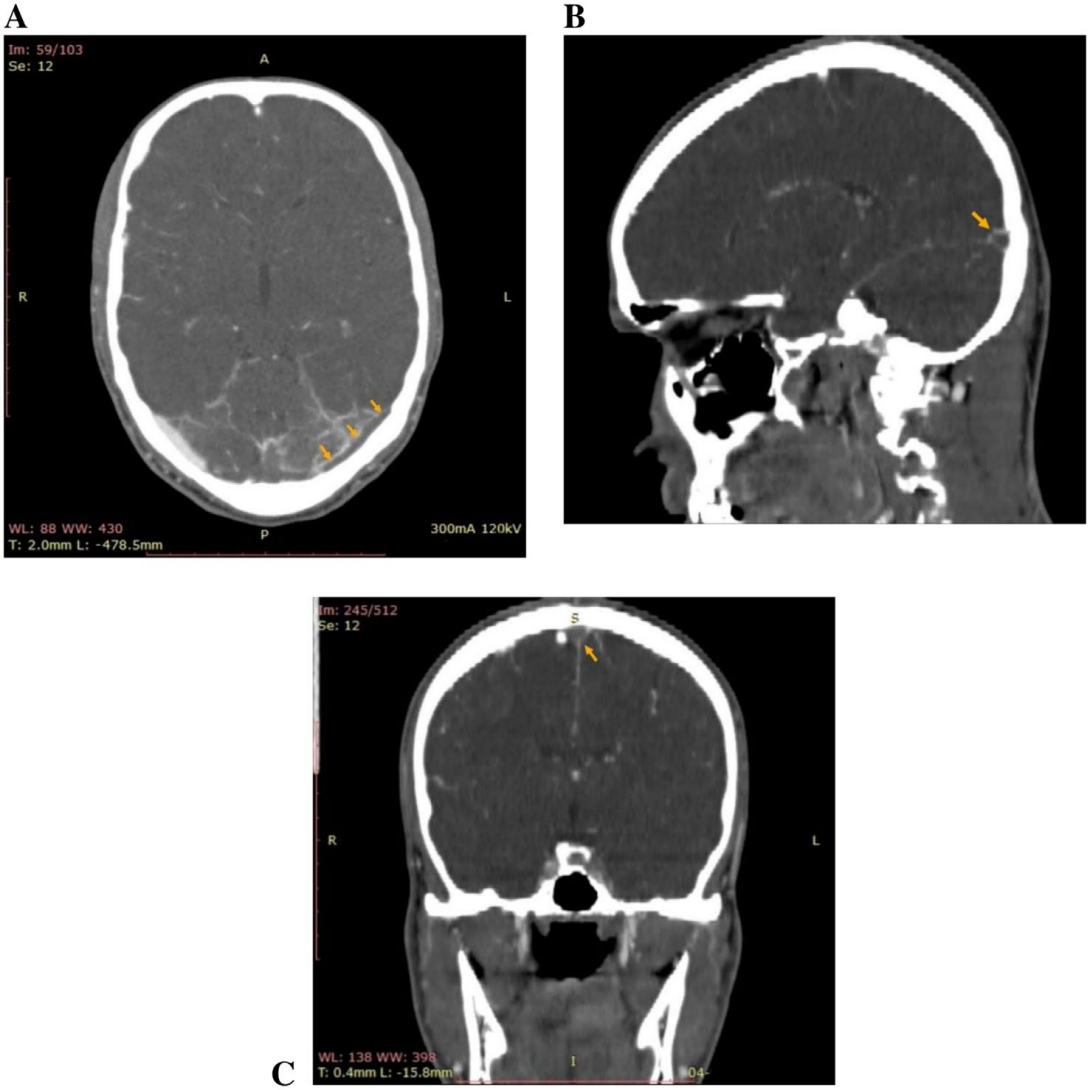
CT cerebral venography (selective images) showing hypodense filling defects, consistent with extensive dural venous sinus thrombosis. Axial view (A) demonstrating thrombosis in the left transverse sinus (yellow arrows). Sagittal view (B) demonstrating thrombosis at the confluence of sinuses (yellow arrow). Coronal view (C) demonstrating thrombosis in the superior sagittal sinus (yellow arrow), the classic ‘empty delta sign.’.

CT cerebral angiography and venogram demonstrated multiple hypodense filling defects noted within the superior sagittal sinus with extension into the bilateral transverse sinuses, left sigmoid sinus, and internal jugular vein. Similarly, a hypodense filling defect was noted within the straight sinus and inferior sagittal sinus, consistent with extensive CVST (Figure 1). Additionally, altered gyriform hypodensities in the left parieto‐occipital region were suggestive of a hemorrhagic venous infarct. Transthoracic echocardiography (TTE) was unremarkable (EF = 60%).

Clinical suspicion for the CVST was made on a constellation of clinical features (progressive headache with vomiting and positional variation), and neuroimaging findings demonstrating dural venous sinus thrombosis confirmed it. Because the patient lacked any traditional risk factors for thrombosis, the condition was considered most likely temporally associated with dengue virus‐induced post‐infectious prothrombotic state; hence, a working diagnosis of post‐dengue CVST was made.

The patient was admitted and managed according to the most recent international standard guidelines for CVST. Immediate parenteral anticoagulation remains the cornerstone of therapy for CVST as dictated by the 2024 American Heart Association/American Stroke Association (AHA/ASA) Scientific Statement as well as the 2017 European Stroke Organization (ESO) clinical practice guidelines for the management of CVST. This applies in the presence of a secondary hemorrhagic venous infarction [8, 9]. Thus, therapeutic anticoagulation was initiated rapidly with subcutaneous low—molecular—weight heparin (enoxaparin, 60 mg daily). The patient's recovering platelet levels provided a safe therapeutic window to initiate enoxaparin, which was aimed at preventing thrombus propagation and facilitating venous recanalization.

In view of cortical involvement and statistically elevated risk of seizure secondary to hemorrhagic venous infarction, intravenous levetiracetam (500 mg twice daily) was administered for seizure prophylaxis due to its safety profile and minimal drug interactions. While seizure prophylaxis is not recommended in all CVST patients. But recent evidence suggests that the presence of supratentorial parenchymal lesions, as in our patient, strongly and independently predicts early acute symptomatic seizures [10].

Supportive treatment included intravenous paracetamol, 1 g Q8H as needed for analgesia, along with adequate intravenous hydration. In the setting of extensive cerebral venous outflow obstruction, the baseline intracranial pressure (ICP) is intrinsically elevated. Thus, oral lactulose (15–30 mL four times daily) was prescribed to prevent constipation and reduce straining. This helps prevent Valsalva maneuver to prevent an acute, transient rise in ICP. A rise in ICP during straining could otherwise provoke hematoma extension and increase chances of localized ischemia [9, 11].

### 2.3 Conclusions and Results (Outcome and Follow‐Up)

The patient remained hemodynamically stable throughout hospitalization and showed rapid, progressive signs of clinical improvement. He was discharged (day 15) on the following oral medications: Rivaroxaban 15 mg Q12H for 3 weeks, followed by 15 mg once daily for 3 months, and levetiracetam 500 mg Q12H for 3 months, along with painkillers as needed. This shift from subcutaneous low‐molecular‐weight heparin to oral factor Xa inhibitor is in line with existing literature, which reports that factor Xa inhibitors have comparable efficacy to traditional vitamin K antagonists for outpatient treatment of CVST [10]. The patient was also counseled to report to the hospital emergency unit in case of any emergency. Regular follow‐up visits to the outpatient department were arranged for reassessment and further management. Over the following months, the patient remained clinically stable, and the treatment plan was completed as planned. The clinical timeline of this case has been summarized in Table 2.

**TABLE 2:** Chronological timeline of clinical events, investigations, and management.

Time frame		Event
Day 1–8	Presentation	A young male presented with fever and generalized body aches.
	Shortly thereafter	Investigations revealed thrombocytopenia with a positive NS1 antigen test.
	Diagnosis	*Dengue fever*, in accordance with endemic patterns.
	Following diagnosis	Managed conservatively, with no reported complications. Platelet levels showed improvement (94 × 10^9^/L on Day 1; 111 × 10^9^/L on Day 5).
Day 9–10		Patient develops a mild, frontal headache, later becoming holocranial. It was not responding to analgesics and was associated with multiple episodes of vomiting.
Current presentation (Day 11)		Presents to us with complaints of headache, vomiting and dizziness.
Shortly after the presentation		Investigations revealed thrombocytopenia and deranged transaminases. CT cerebral angiography and venogram were consistent with CVST.
Working diagnosis		*Post‐dengue CVST*.
Following diagnosis		Initiated on anticoagulation and seizure prophylaxis. Additional treatment included analgesics, IV fluids, and a laxative.
Subsequent course (day 12–14)		Remained hemodynamically stable and showed significant clinical improvement.
Day 15		Discharged on oral medications, including an anticoagulant and antiepileptic, along with analgesics on a need basis
Follow‐up visits		Remained clinically stable, and the treatment plan was executed as planned

## 3 Discussion

This case features a 29‐year‐old male presenting with CVST 11 days after being diagnosed with dengue fever. Although CVST can occur in a number of conditions, its incidence in viral illnesses, such as dengue, is infrequent [6]. Arterial strokes have occasionally been reported in dengue; CVST, in comparison, remains an uncommon and often underrecognized complication, with only a few cases documented in the literature, including the current one. Dengue fever is well known for its hemorrhagic complications; however, dengue‐linked thrombosis has been increasingly recognized in recent years [2]. The coexistence of thrombocytopenia and thrombosis in such cases presents both diagnostic and therapeutic challenges, particularly concerning the use of anticoagulation [12]. This paradox highlights the importance of recognizing dengue not only as a hemorrhagic illness but also as a potential trigger of thrombotic complications.

The mechanism of dengue‐associated thrombosis is complex and depends on the stage of illness. During acute and critical phases (days 1–7), dehydration, hemoconcentration, and plasma leakage can increase blood viscosity and lead to venous stasis, which predisposes to thrombosis [13]. However, in the recovery phase (> day 7), endothelial dysfunction and immune‐mediated thromboinflammation play a more dominant role. Potential mechanisms suggest that the virus can directly infect and activate endothelial cells, interfering with the thrombomodulin–thrombin–protein C anticoagulant pathway and reducing activated protein C activity. Simultaneously, natural anticoagulants such as protein S and antithrombin III may become deficient due to consumption and capillary leakage. Collectively, these changes may shift the body's normal hemostatic balance toward a transient hypercoagulable state, even in the presence of thrombocytopenia. In addition, persistent immune activation and cytokine release contribute to a prothrombotic milieu. This process, often termed “*thromboinflammation*,” may extend beyond the acute phase of infection, resulting in a transient hypercoagulable state [13]. In the present case, the onset of headache secondary to CVST approximately 8 days after the initial infection places the patient in the early recovery phase, a period increasingly recognized for persistent vascular and immunological dysregulation [14]. However, in our case, only a temporal association can be stated, as complete exclusion of alternative prothrombotic etiologies was necessary for definitive diagnosis. Unfortunately, this could not be achieved due to limited resources and unavailability of some investigations.

The diagnosis of CVST after recent dengue infection can be challenging, as headache is the most common presenting symptom, which is nonspecific and may be attributed to the underlying viral illness. The lack of focal neurological deficits, as seen in our case, may further delay clinical suspicion [15]. Therefore, a high index of clinical suspicion should be maintained, particularly in patients presenting with persistent or atypical headache following dengue infection. Neuroimaging plays an important role in establishing a definitive diagnosis, with magnetic resonance imaging combined with venography (MRI/MRV) considered the gold standard. However, MRV has certain limitations, particularly in detecting cortical vein thrombosis and in differentiating congenital hypoplasia of venous sinuses from true thrombotic occlusion. In such circumstances, especially where resources are limited, CT venography represents a reliable and rapid alternative and was therefore utilized in our patient. It demonstrates characteristic imaging features, including venous sinus filling defects, wall enhancement, and collateral venous drainage. Despite these advantages, it is less sensitive for detecting small or deep venous thromboses. Furthermore, its use is associated with exposure to ionizing radiation as well as potential contrast‐related risks [16].

The management of CVST in patients with recent dengue infection presents a therapeutic dilemma, requiring a careful balance between thrombotic and hemorrhagic risks. Clinicians often hesitate to start anticoagulation in patients with recent dengue infection, especially when hemorrhagic venous infarction is present. For instance, Sharma et al. proposed to treat dengue‐associated CVST with intravenous hydration alone due to massive intracerebral bleed in their patient secondary to anticoagulation [17]. However, modern neurocritical approach refutes this proposal and suggests that careful assessment of hematological parameters can help guide safe treatment decisions [16]. In this case, the patient's platelet count had recovered to 135 × 10^9^/L at presentation, representing a safe therapeutic window for anticoagulation. Current, high‐level evidence explicitly codified in the 2024 AHA/ASA guidelines and the 2017 ESO/EAN clinical practice guidelines strongly supports anticoagulation as the mainstay of treatment, even in the presence of hemorrhagic venous infarction, provided there are no absolute contraindications [8, 9]. We initiated therapeutic anticoagulation with low‐molecular‐weight heparin, leading to favorable clinical improvement without bleeding complications. This is consistent with previously reported cases, further supporting the safety and efficacy of anticoagulation in similar clinical scenarios [15].

Supratentorial and hemorrhagic lesions are recognized as major predisposing factors leading to acute as well as late seizures in patients with CVST. In view of possible seizure risk, a prophylactic antiepileptic was given to our patient. Levetiracetam was selected due to its favorable safety and efficacy [18, 19]. As part of the supportive therapy, the patient received lactulose to prevent straining efforts. It is an important neuroprotective mechanism, also applicable to CVST patients, as straining increases intracranial pressure more in the presence of cerebral venous outflow obstruction. This can exacerbate the risk of hematoma expansion and secondary ischemic injury [9, 11]. During the hospitalization and follow‐ups, our patient showed gradual clinical improvement and was discharged in stable condition, highlighting the importance of early recognition, prompt initiation of appropriate therapy, and comprehensive supportive management in optimizing clinical outcomes.

Furthermore, our transition to oral factor Xa inhibitor (Rivaroxaban) upon discharge reflects the latest paradigm shifts in vascular neurology. Recent trial data confirm the safety of factor Xa inhibitors in the management of CVST. They are now considered non‐inferior to traditional vitamin K antagonists [10].

### 3.1 Limitations

This case report has certain limitations despite its clinical relevance. As a single‐patient observation, it cannot establish a definitive causal relationship between recent dengue infection and CVST, and only a temporal association can be inferred. In addition, a complete evaluation for underlying prothrombotic conditions was not done, including thrombophilia workup such as protein C, protein S, antithrombin III levels, factor V Leiden mutation, antiphospholipid antibodies, and homocysteine levels, due to financial constraints, which is a key concern in resource‐limited settings. Furthermore, neuroimaging was based on CT findings without confirmatory MRV due to a lack of availability. Future research should focus on these limitations to provide greater insight into dengue‐associated CVST.

## 4 Conclusion

CVST occurring in temporal association with dengue is a rare but potentially life‐threatening complication that may occur even after apparent clinical recovery. Clinicians should maintain a strong clinical suspicion for CVST in patients presenting with persistent, severe, or progressively worsening headache within 1–2 weeks following dengue diagnosis. Early neuroimaging and prompt initiation of anticoagulation, once platelet counts have safely recovered, are essential to prevent catastrophic neurological outcomes and ensure a favorable prognosis.

## Author Contributions

**Zain Ul Abedeen:** conceptualization, methodology, data curation, investigation, project administration, writing – original draft, writing – review and editing. **Waqar Khan:** methodology, validation, visualization, writing – review and editing, writing – original draft. **Zia Ullah:** methodology, visualization, validation, writing – review and editing, data curation. **Waqar Iqbal Khan:** conceptualization, methodology, visualization, writing – review and editing, writing – original draft. **Naeem Ullah:** conceptualization, supervision, project administration, data curation, validation, writing – review and editing. **Abad Ur Rehman:** writing – review and editing, data curation, visualization, validation.

## Funding

The authors have nothing to report.

## Ethics Statement

The authors have nothing to report. It has been prepared in accordance with CARE guidelines for writing case reports, as per standard practice and journal requirements.

## Consent

Written informed consent was obtained from the patient for publication of this case report and accompanying images.

## Conflicts of Interest

The authors declare no conflicts of interest.

## Data Availability

The data that support the findings of this study are available on request from the corresponding author. The data are not publicly available due to privacy or ethical restrictions.
